# Artificial Vision Systems for Fruit Inspection and Classification: Systematic Literature Review

**DOI:** 10.3390/s25051524

**Published:** 2025-02-28

**Authors:** Ignacio Rojas Santelices, Sandra Cano, Fernando Moreira, Álvaro Peña Fritz

**Affiliations:** 1 Doctorate in Smart Industry, Pontificia Universidad Católica de Valparaíso, Avenida Brasil 2141, Valparaiso 2370688, Chile; benito.rojas.s01@mail.pucv.cl; 2 School of Informatics Engineering, Pontificia Universidad Católica de Valparaíso, Avenida Brasil 2241, Valparaiso 2370688, Chile; sandra.cano@pucv.cl; 3 REMIT (Research on Economics, Management and Information Technologies), IJP (Instituto Jurídico Portucalense), Universidade Portucalense, Rua Dr. António Bernardino de Almeida, 541-619, 4200-072 Porto, Portugal; 4 IEETA (Instituto de Engenharia Electrónica e Telemática de Aveiro), Universidade de Aveiro, 3810-193 Aveiro, Portugal; 5 School of Construction and Transportation Engineering, Pontificia Universidad Católica de Valparaíso, Avenida Brasil 2147, Valparaiso 2370688, Chile; alvaro.pena@ucv.cl

**Keywords:** fruit classification, quality inspection, quality control, computer vision, image processing, artificial vision, deep learning, artificial intelligence

## Abstract

Fruit sorting and quality inspection using computer vision is a key tool to ensure quality and safety in the fruit industry. This study presents a systematic literature review, following the PRISMA methodology, with the aim of identifying different fields of application, typical hardware configurations, and the techniques and algorithms used for fruit sorting. In this study, 56 articles published between 2015 and 2024 were analyzed, selected from relevant databases such as Web of Science and Scopus. The results indicate that the main fields of application include orchards, industrial processing lines, and final consumption points, such as supermarkets and homes, each with specific technical requirements. Regarding hardware, RGB cameras and LED lighting systems predominate in controlled applications, although multispectral cameras are also important in complex applications such as foreign material detection. Processing techniques include traditional algorithms such as Otsu and Sobel for segmentation and deep learning models such as ResNet and VGG, often optimized with transfer learning for classification. This systematic review could provide a basic guide for the development of fruit quality inspection and classification systems in different environments.

## 1. Introduction

The fruit industry is an essential pillar of the global economy and a fundamental component of the world’s food supply [1]. The growing demand for high-quality fresh products has driven the need to implement efficient inspection and control systems that guarantee the safety and absence of contaminants in fruits intended for consumption [2]. Accurate classification and early detection of defects and contaminants not only ensure consumer satisfaction but also align with international food safety regulations [3].

The application of computer vision in fruit inspection and quality control has emerged as an advanced technological solution to address these challenges [4]. In the field of precision agriculture, these systems are used during the harvesting stage to determine the optimal picking time, ensuring that fruits achieve their maximum nutritional and organoleptic quality [5]. Furthermore, computer vision enables the precise recognition and localization of fruits on the tree, facilitating harvest automation through robotic pickers. These systems can identify the maturity, size, and position of the fruit, allowing for selective harvesting that minimizes fruit damage and enhances the efficiency of automated harvesting [6,7]. In the packaging and sorting stages of fruit-exporting companies, artificial vision allows for more efficient and precise selection, reducing the margin for human error and speeding up production processes [8]. In addition, in supermarkets, these technologies can help the consumer by evaluating the freshness and quality of fruits in real time, facilitating more informed purchasing decisions [9]. A study proposed by [10] investigated methods and challenges associated with fruit size measurement through artificial intelligence, addressing detection and segmentation techniques. The study concludes that deep learning methods have significantly outperformed traditional techniques, although challenges remain concerning the variability of lighting conditions and the lack of specialized public datasets. The authors conducted a review specifically focused on systems applied to orchards, highlighting the importance of technologies that facilitate automated fruit harvesting. Their work focuses on developing mechanisms capable of operating in open-field conditions, where factors such as light variability and the presence of vegetation complicate the precise detection of fruits.

Traditionally, manual inspection has been the primary method for assessing fruit quality, but this process is time-consuming, subjective, and prone to human error. To address these limitations, researchers have explored the use of artificial vision systems for automated fruit quality inspection. Artificial vision systems, powered by advanced image processing and machine learning algorithms, can overcome these limitations and offer a more robust and efficient solution for quality inspection. These systems can perform real-time defect detection, classify defects, and provide automated decision-making capabilities, thereby improving the overall quality control process [11]. In [12], the impact of deep learning in fruit image analysis, addressing various approaches and model architectures, such as classical CNNs, R-CNN, and YOLO. A relevant aspect mentioned in this study is the challenges associated with the lack of data, the complexity of labeling, variations in fruit characteristics, and computational efficiency. The authors also emphasize the importance of multispectral data integration and knowledge transfer between species to improve the robustness of systems in dynamic environments. This work represents a significant contribution by identifying trends and suggesting future lines of research, such as the integration of models in real agricultural systems and the development of advanced data augmentation techniques to overcome limitations in reduced datasets.

Although there are significant advances in this field and the use of different techniques and approaches for fruit inspection and sorting, there are difficulties in identifying which are the most efficient and reliable hardware and software configurations. The speed and accuracy of the algorithms are critical factors, especially when handling large amounts of images in real time, which highlights the importance of developing systems capable of processing data quickly without compromising accuracy. This capability is fundamental for the successful adoption of these systems in industrial and commercial environments.

Main contribution with this study is to identify key trends, methodologies, and technologies used in this field such machine learning, deep learning, and image-processing techniques. In addition, this study provides a detailed understanding of the current state of research in artificial vision systems for inspection and classification. This study could be an alternative to help researchers quickly understand which techniques are most suitable for specific tasks (e.g., defect detection, quality grading). This study also highlights the importance of preprocessing techniques, segmentation algorithms, feature extraction, and classification methods in the development of effective computer-vision-based quality inspection systems. This knowledge is crucial in guiding the development of more advanced and robust artificial vision systems for fruit inspection and classification.

This article is structured as follows: Section 2 presents the objectives of this systematic literature review and defines its research questions. Section 3 explains the methodology used for the literature search, which is guided by the Preferred Reporting Items for Systematic reviews and Meta-Analysis (PRISMA) guidelines [13]. Section 4 provides the results found in the articles selected. Section 5 discusses the reviewed studies. Finally, Section 6 offers the conclusions.

## 2. Objectives

The objective of this systematic literature review is to examine the research that has been carried out on inspection of quality of fruits using vision artificial systems

The objective is to synthesize the current research to increase our understanding of algorithms and techniques that must follow artificial vision systems for quality inspection of fruit. This systematic literature review (SLR) aims to answer three research questions that are derived from the previously mentioned problems:

○ RQ1: What are the application fields where it is required to classify and inspect fruit using artificial vision?
○ RQ2: What are the typical hardware configurations in machine vision systems used for image acquisition in fruit classification and inspection?
○ RQ3: What are the most used image-processing algorithms and techniques in fruit classification and inspection?

## 3. Methodology

**Figure 1:**
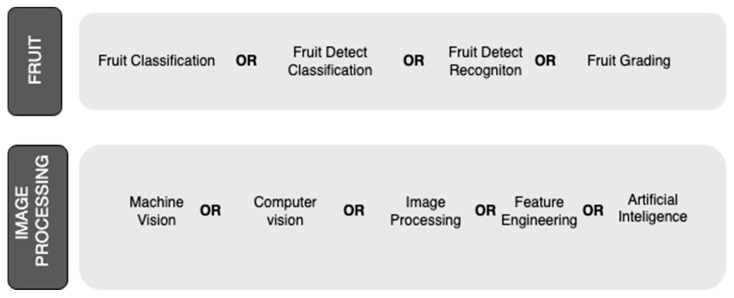
Search strategy used in databases.

To address the research questions, a thorough plan for the search and selection of relevant literature was carried out following the PRISMA guidelines [13]. The search for articles was carried out in two of the most important scientific databases: Web of Science and Scopus. Key search terms related to the topic of the review were defined, ensuring adequate coverage of computer vision techniques and applications in fruit classification. The terms used were as follows: “Fruit Classification”, “Fruit defect classification”, “Fruit defect recognition”, “Fruit grading”, “Machine Vision”, “Computer vision”, “Image processing”, “Artificial vision”, “Feature Engineering”, and “Artificial Intelligence”. These terms can be divided into two interconnected groups: the first refers to aspects related to fruit, and the second to computer vision and image processing. The scheme presented in Figure 1 shows the search strategy that links both groups. The identification of these key terms was performed by searching in the titles, abstracts, and keywords of the articles.

The initial search generated a total of 505 articles combining WoS and Scopus. Subsequently, additional database filters were applied to refine the results: articles published between 2015 and 2024 were selected, the document type was restricted to “Article”, the source to “Journal”, the language of publication to “English”, and the availability of the articles, including in this review only those categorized as open-access. This allows for focusing on the most recent literature and previous studies relevant to computer vision applied to fruit classification and quality inspection. After applying these filters, 310 documents were removed. Then, 126 duplicate articles were eliminated using the Mendeley tool, leaving 69 articles. In the screening stage, a manual review of the titles and abstracts was performed to assess their eligibility, excluding those papers that did not meet the inclusion criteria set out in Table 1. One article that could not be retrieved was excluded. Finally, after a complete texts review, one article was excluded by applying the exclusion criterion (EC1) and one article by applying the exclusion criterion (EC2). As a result, 56 articles were selected for the final review.

**Figure 2:**
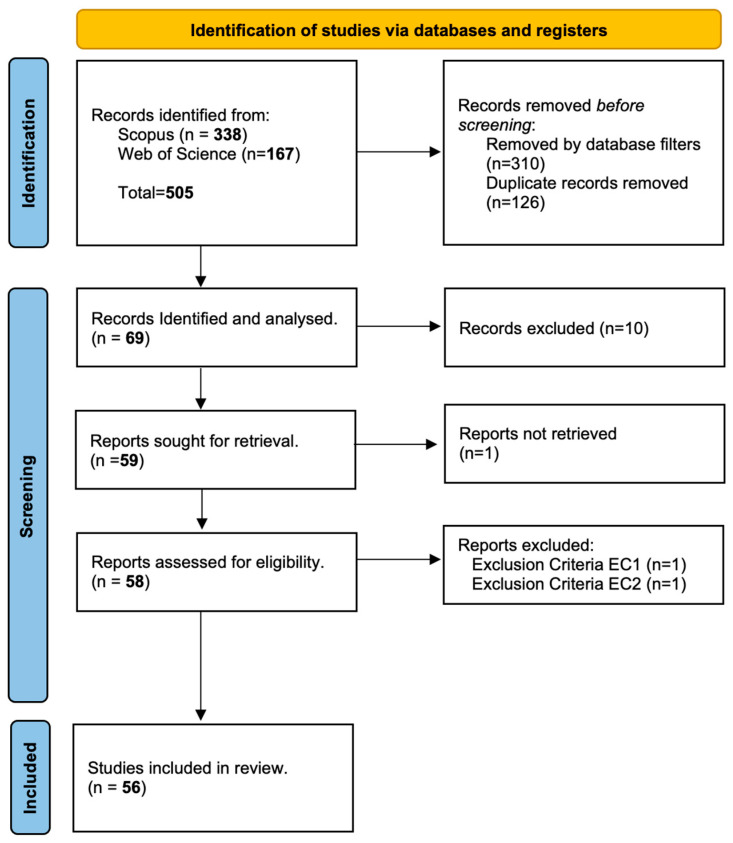
Article selection scheme according to PRISMA [13].

Figure 2 represents the flow chart of the studies selected according to the PRISMA statements.

## 4. Results

The search of the databases resulted in a total of 56 articles. Finally, 56 articles published between 2015 and 2024 were selected after considering the inclusion and exclusion criteria. The selected articles allowed us to answer the study questions.

### 4.1. Data Synthesis

Table 2 shows a summary of articles selected, which describes a set of aspects found for each study.

### 4.2. General Articles Characteristics

The general characteristics of the selected studies are presented below, which allows for contextualizing the temporal, geographical, and thematic distribution of research in artificial vision applied to the classification and quality control of fruits.

**Figure 3:**
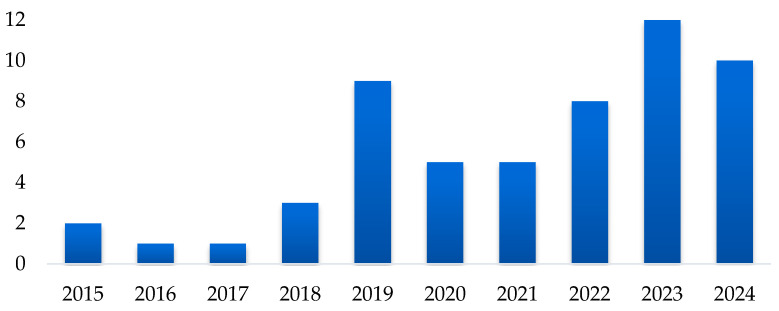
Distribution of studies in the time analyzed.

A. Distribution of studies in the time analyzed: Approximately 70% of the studies were published between 2020 and 2024, reflecting a significant growth in recent research. This increase may be related to advances in computer vision techniques and the growing interest in applying these technologies to agriculture and the food industry to optimize fruit sorting and quality control processes. Figure 3 shows the temporal evolution of research.

**Figure 4:**
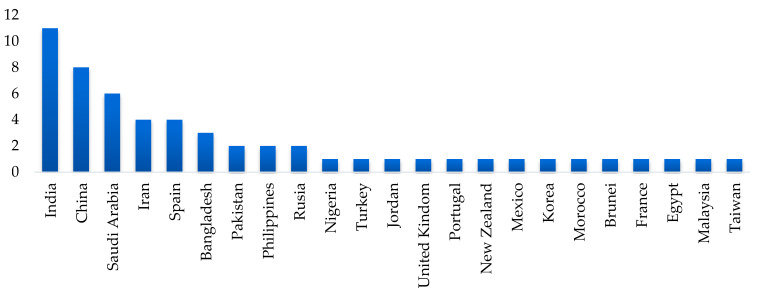
Studies distribution by geographical location.

B. Productivity by Country: Figure 4 shows studies distribution by geographical location. The leading countries in terms of the number of publications in this field are India, with 11 studies, followed by China with 9 studies, and Spain and Iran with 4 publications each. This suggests a particular interest in these regions, possibly due to their relevance in fruit production and export, as well as the presence of active research groups in agricultural technology and food processing.

**Figure 5:**
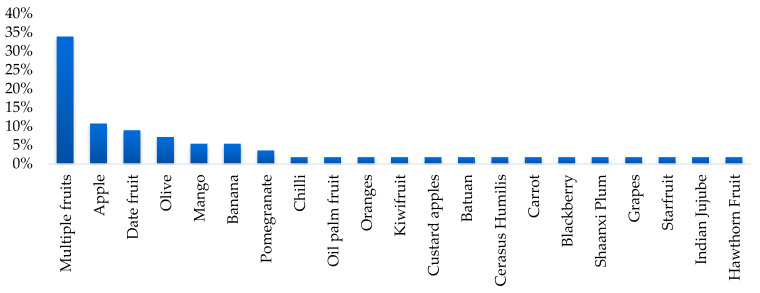
Percentage distribution according to fruit type.

C. Product Type: The relevance of certain types of fruit varies by region, but there is significant interest in classifying multiple types of fruit or varieties. This type of work represents 35% of the studies. In terms of specific fruits, apples stand out, with 10.5% of the studies, followed by dates, with 8.8%. The distribution shown in Figure 5 indicates a trend towards the creation of versatile systems that can be applied to different fruits, as well as a specialization in products of high commercial relevance.

D. Type of Dataset Used: Most studies (74%) used proprietary datasets, created specifically to develop and test their classification models. In total, 24% of the studies used benchmark datasets, while approximately 2% used a combination of benchmark and proprietary datasets. This predominance of custom datasets indicates the need to adapt the models to specific characteristics of each type of fruit or capture condition, although the use of benchmark datasets is beginning to consolidate as a practice to ensure the reproducibility and comparability of the results. Table 3 shows the datasets used in the articles under study, including information on the number of images taken from each one for training the classification algorithms.

In the reviewed studies, a variety of classification objectives are observed, reflecting the specific needs of each application in artificial vision for the fruit industry. The most common objective was classification by fruit type, present in 32% of the studies, highlighting its importance for tasks such as automation in production lines and fruit recognition at points of consumption. The second most frequent objective was the assessment of the state of ripeness, with 23% of the studies focusing on determining levels of ripeness for applications such as automated harvesting, post-harvest quality control, and fruit selection according to commercial standards.

**Figure 6:**
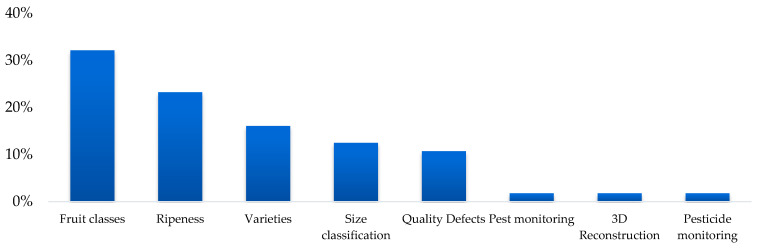
Percentage distribution by classification objective.

E. Classification by type: Classification by type of variety represented 16% of the studies, a particularly relevant task for products with multiple commercially significant varieties, such as apples, dates, or mangoes, where varietal characteristics directly affect commercial value and consumer preferences. These objectives reflect how classification applications vary by context, from early stages of production to direct interaction with the final consumer. Figure 6 shows the percentage distribution of each type of application.

### 4.3. Answering the Research Questions

The objective of the abstraction was to respond to the following questions:

Question 1. What are the application fields where it is required to classify and inspect fruit using artificial vision?

Artificial vision is applied in three main areas within the fruit industry:

A. Orchard: At this stage, machine vision systems are used to automate the harvesting and sorting of fruit directly in the field. These systems are useful for identifying the fruit classes [32,38,43], assessing maturity levels [23,24,28,30,51], pest monitoring [49], and pesticide monitoring [65]. In addition, their implementation makes it possible to address the challenges related to labor shortages in agricultural activities.
B. Fruit-processing industries: In industrial processing lines, machine vision is used for tasks such as varieties detection [14,20,50,54,64,70], fruit classes [18,19,35], ripeness level [21,25,27,55,57,66], size classification [22,26,29,33,36,60,63], and quality defects sorting [34,42,52,56,62,67]. This use stands out for its ability to reduce human error, increase inspection speed, and improve consistency in product quality.
C. Retail or Final Consumption Points: In this emerging area, artificial vision systems are designed to assist the consumer or distribution chains in assessing freshness [48], [53], identifying varieties [17,58,61], and detecting the type of fruit [15,16,31,37,39,40,41,44,45,46,59,69]. Recent advances have allowed for technologies integrated into smartphones to classify fruit in real time, facilitating informed purchasing decisions.

Question 2. What are the typical hardware configurations in machine vision systems used for image acquisition in fruit classification and inspection?

Hardware configurations vary depending on the application context, but some main trends are identified:

A. Image capture:

The reviewed studies show that RGB (Red, Green, Blue) cameras are the most used sensor type in fruit classification, accounting for 84% of applications (Table 4.) RGB cameras enable effective detection of product variety and distinguish between different types of fruit, as well as identifying quality defects, such as ripeness and presence of rot. This type of sensor is suitable for detecting visual characteristics in the visible-light range, facilitating basic quality analysis and fruit sorting.

On the other hand, a set of studies focus their efforts on the use of hyperspectral cameras [34,49,62], which provide a broader spectrum of information and allow for the detection of insect contamination, the presence of pesticides, and surface damage that are not perceptible with RGB sensors. Hyperspectral technology is especially useful in applications that require a detailed assessment of the surface and composition of the fruit to ensure product safety and quality.

The use of smartphones as sensors for image acquisition has gained importance in recent years, especially in studies aimed at fruit recognition at final consumption points [17,27]. These smartphone-based models allow for the identification of the fruit type, as well as the evaluation of its state of ripeness and detection of possible rot, making this technology accessible to end users.

B. Acquisition Conditions:

Regarding acquisition conditions, it is observed that 61% of the studies use ambient light to capture images, mainly in sorting applications in orchards or at final consumption points, where light variability is a natural condition. This type of light requires that the artificial vision algorithms adapt to light fluctuations, since ambient lighting can affect the accuracy of detection and sorting.

**Figure 7:**
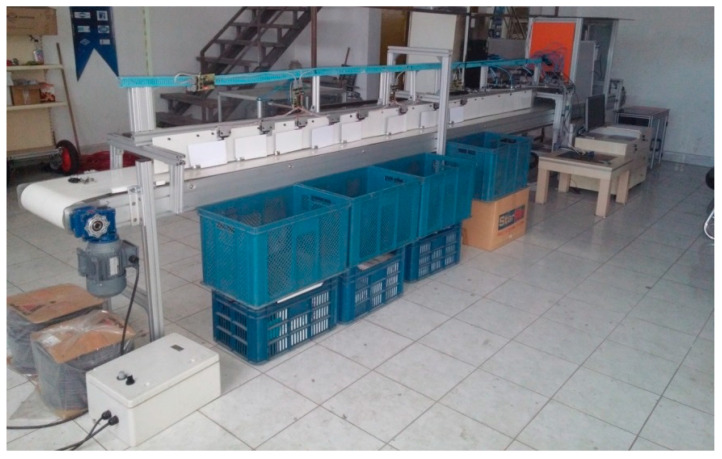
Carrot-sorting machine [33].

In studies related to the fruit-processing industry, the use of LED lights predominates, which provide controlled and stable lighting. This type of light allows for greater consistency in images, which is crucial for industrial applications that require high precision and consistency in quality assessment. In addition to the lighting system, in projects related to the food industry, it is important to have a transport system that allows for in-line sorting, managing continuous production volumes for efficient processing (Figure 7).

In addition, the assembly of cameras and lighting systems is carried out within a dome that allows the sorting system to be isolated from external agents. This dome provides uniform lighting, which reduces the appearance of shadows and reflections to a minimum, thus improving the quality of the images obtained and the accuracy of classification.

Studies using hyperspectral cameras predominantly use halogen light. The need to record images in the infrared range, where some quality defects are more visible, makes halogen light a suitable choice, as it provides a broad spectrum that facilitates the detection of quality details that are not evident in the RGB color space.

C. Capture speed:

Capture speed is not a critical factor in the studies reviewed, as most of them work under static conditions for the generation of datasets or for classification in the laboratory. This means that image capture is carried out slowly, without the need for a high capture speed, which minimizes the influence of this factor on the results obtained.

Question 3. What are the most used image-processing algorithms and techniques in fruit classification and inspection?

The most used techniques range from traditional approaches to modern deep learning models:

A. Preprocessing:

The preprocessing stage is essential in fruit classification using computer vision, as it allows for images to be optimized before being analyzed by segmentation and classification algorithms. Depending on the type of study and the environment in which the images are captured (such as orchards, processing lines, or supermarkets), significant differences may arise in terms of size, lighting, resolution, and quality of the images. These variations require specific adjustments to ensure that the images are consistent and suitable for analysis.

- Image filtering and enhancement

**Figure 8:**
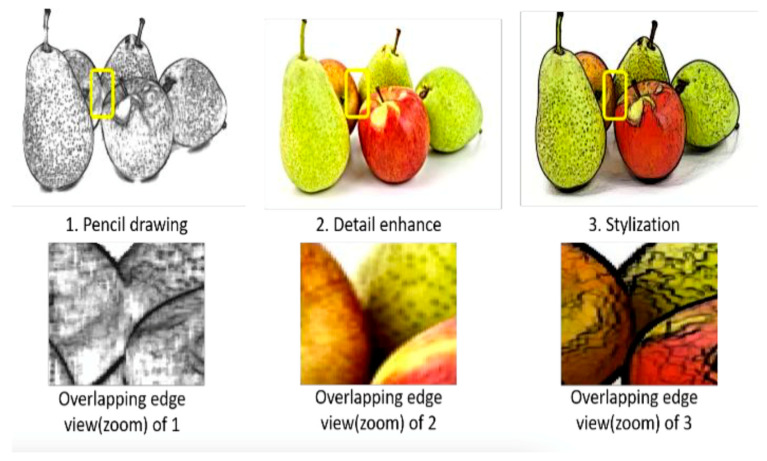
Stylization filter applied to improve object detection [61].

Studies show the use of filtering techniques to reduce noise and improve the visibility of objects. In [61], a stylization filter is used (see Figure 8), which allows for drawing a black line on the outline of the object to make a better segmentation later.

Gaussian filtering is one of the most widely applied techniques, allowing images to be smoothed without compromising the relevant details that facilitate classification [21,63,66,68]. Furthermore, image enhancement through contrast and sharpness adjustments is commonly used to highlight distinctive features of fruits, such as defects or variations in texture, which facilitates more accurate and effective feature extraction later on [23].

- Color adjustment and lighting correction

The reviewed studies highlight its importance in environments where lighting is variable, such as in orchards or points of consumption. Researchers have used the RGB value normalization techniques and transformations in alternative color spaces, such as HSV (Hue, Saturation, Value) or CIE L*a*b*, to compensate for light fluctuations and improve consistency in images [24,25]. Also, in low-light conditions, in [23], image fusion methods were applied, combining data from the visible and infrared spectrum to achieve greater contrast and visual quality between the fruit and the background composed mainly of vegetation.

- Geometric transformations

According to the information extracted, the main objective of applying geometric transformations in the reviewed studies is to increase the amount of available data through data augmentation. Studies such as [21,25,41,70] use rotation techniques, saturation adjustment, brightness adjustments, hue modification, as well as reflection and mirroring.

**Figure 9:**
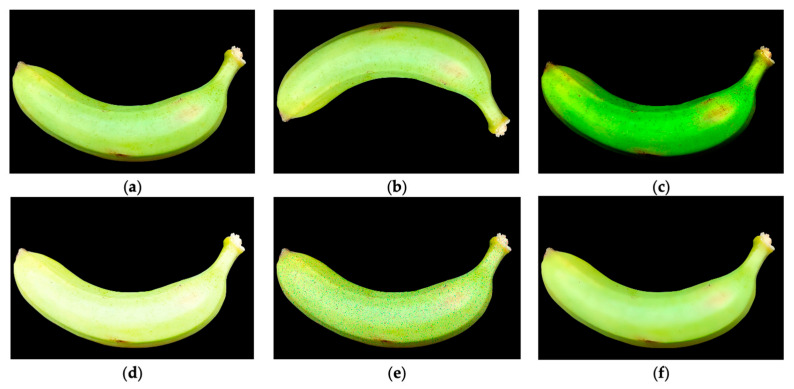
Different transformations applying data augmentation: (**a**) original image, (**b**) rotation, (**c**) darkening, (**d**) brightening, (**e**) pretzel, and (**f**) blurring [21].

Data augmentation is essential in image analysis because it allows to artificially expand the size of the dataset without the need to collect new images, which is especially useful when the amount of available data is limited. By introducing variations in the original images (see Figure 9), such as changes in orientation, color, and brightness, the computer vision model is exposed to a more diverse set of data, which improves its generalization ability and reduces the risk of overfitting [21]. This is critical for the model to perform well in unseen conditions and to be more robust against variations in the environment, lighting, and object position in real-world scenarios.

A. Segmentation:

The next step after image capture and preprocessing is segmentation, a key stage whose main objective is to separate the object of interest, such as a fruit, from the image background. This background can vary from uniform colors to complex environments with vegetation, accessories or other elements. As evidenced in studies reviewed, the need for segmentation depends on the type of study and its application. For example, in cases such as studies [25,28], segmentation is not carried out; instead, the process goes directly from preprocessing to feature extraction. This occurs especially in works related to classification at points of consumption or retail, where the objective is to identify the type of fruit (for example, distinguishing between an apple and a banana).

On the other hand, in most studies, segmentation is necessary to individualize each fruit, separating it from the background, and thus perform a more accurate classification without influences from other objects. In some cases, such as in studies [17,21,25], uniform black or white backgrounds are used to generate a high contrast, facilitating the application of segmentation algorithms. However, in other studies [59,66] where fruits are found in uncontrolled backgrounds, it is necessary to resort to more advanced techniques, such as adaptive thresholding, a method that dynamically adjusts the threshold value to segment regions of interest in images with lighting variations or complex backgrounds.

- Segmentation algorithms

Segmentation is a crucial stage in computer vision systems, and the algorithms applied depend on the characteristics of the images and the objectives of the analysis. In the reviewed studies, multiple techniques are identified to separate objects from the background, many of which generate binarized masks that facilitate subsequent analysis. Some of the most used algorithms and methods, as well as complementary techniques, are described below.

  ○ Sobel Filter [17,27]: It is an edge detection technique that calculates the derivative of pixel intensity in horizontal and vertical directions, highlighting areas where sharp changes in intensity occur. This method uses two convolutional masks (kernels), one for each direction, and combines the results to obtain a gradient image. It is useful for identifying contours in images where the edges are sharp and well defined, providing key information for segmenting objects such as fruits. Although it is efficient, its performance can be affected in noisy images, so it is often combined with pre-filtering techniques to improve the quality of the results.
  ○ Canny Filter [22,33,57]: This algorithm is a more advanced technique for edge detection. It works in several stages: First, it applies a Gaussian filter to smooth the image and reduce noise; then, it calculates intensity gradients to identify areas with pronounced changes. Next, it uses a “non-maximum suppression” process to refine the detected edges and remove spurious lines. Finally, it applies a double threshold to identify strong and weak edges, connecting weak ones to strong ones if they are related. The Canny filter is especially effective on complex images, as it generates more accurate edges than other techniques.
  ○ Otsu Thresholding [42,46,52,55,64]: Otsu is a threshold-based segmentation technique used to binarize images. This algorithm automatically determines the optimal threshold value by minimizing the intra-class variance and maximizing the inter-class variance. In practical terms, it searches for the ideal cut-off point to separate pixels into two groups: background and object. It is especially useful when the image histogram shows a bimodal distribution, meaning there are two distinct classes (for example, a fruit and its background). Otsu is commonly used on images with uniform illumination and is efficient for applications where an automatic and fast segmentation process is required.
  ○ Mean Shift Clustering [66]: It is a method based on grouping pixels according to their similarity in features such as color or intensity. This algorithm iterates to find the highest densities in the feature space, moving a kernel towards areas with higher density until reaching convergence. It is particularly useful for segmenting images with homogeneous color regions, such as fruits on uniform backgrounds, since it does not require a fixed number of clusters to be specified.
  ○ Watershed Segmentation [20,66,70]: It is based on interpreting the intensity of pixels as a topography where the lowest values represent valleys and the highest, ridges. This method floods the valleys of the image with “water” from marked points, separating regions based on their natural boundaries. It is ideal for segmenting objects that are superimposed or in contact, such as stacked fruit, and allows for obtaining precise contours in complex images. To avoid over-segmentation, it is often combined with preprocessing techniques, such as smoothing and edge detection.
  ○ Combined Applications: In the reviewed studies, it was observed that segmentation techniques are often applied in combination to improve accuracy. The Sobel filter is employed to detect initial contours, which are then refined using the Canny algorithm. Mean Shift Clustering is used to cluster pixels before applying Watershed [66], which reduces noise and improves object separation. In complex or noisy images, these techniques are combined with transformations to color spaces, such as CIE L*a*b* or HSV [27], where chromatic differences between the object and the background are more pronounced, facilitating segmentation. The use of binarized masks not only facilitates background removal but also enables the analysis of physical features, such as measuring longitudinal and transverse axes, calculating area, or identifying specific shapes [26].

In the study [42], two main approaches for segmentation are mentioned:

  ○ Discontinuity-based: Identify abrupt changes in pixel intensity, such as edges or lines. This approach is useful for detecting contours and separating regions with defined boundaries.
  ○ Similarity-based: Groups regions with homogeneous characteristics, such as color intensity or texture. An example of this method is Otsu Thresholding.

- Segmentation Objectives

Segmentation objectives vary depending on the application of the study:

  ○ Object Detection: Identify and isolate the fruit from the background to perform a specific analysis.
  ○ Shape Analysis: Extract the geometry and dimensions of the fruit to evaluate its quality or classify it according to specific standards.
  ○ Color Detection: Identify shades that allow determining the level of ripeness, freshness or presence of defects.
  ○ Defect Detection: Highlight imperfections such as bruises, stains, or physical damage that affect the quality of the fruit.

B. Feature extraction:

Feature extraction is a fundamental stage in classification systems based on image processing, as it allows visual data to be transformed into quantitative descriptors that facilitate the precise and efficient classification of objects. From the articles analyzed, it was found that this stage focuses on identifying attributes such as size, color, texture, shape, and spectral characteristics, depending on the objective of the analysis and the type of sensor used to capture images.

1. *Type of extracted features*

The reviewed studies highlight that the most extracted features include color, texture, shape, and size (Table 3). For example, color and texture are key indicators in detecting ripeness and defects in fruits, while size and shape are essential for assessing the quality, grade, or variety of fruits. In specific research, such as in the case of olives and mangoes, spectral and textural features play a crucial role in variety classification and defect analysis.

2. *Feature Extraction Methods*

Among the methods used for feature extraction, two main approaches stand out: deep learning-based techniques and classical image-processing methods. Both approaches are used to identify and extract key attributes, such as texture, color, shape, and size, depending on the specific objectives of each study.

- Deep Learning-Based Approaches: Deep learning models, such as VGG-16, ResNet, DenseNet, and YOLO, are widely used for feature extraction. These models can identify complex patterns related to texture, color, and shape. In the context of fruit classification, these features are essential for tasks, such as defect detection, quality assessment, and classification by type or variety.
- Use of Color Spaces: The reviewed studies reveal the importance of using different color spaces in feature extraction for fruit classification, as these provide more specific and discriminative representations compared to the standard RGB color space.

In the study on starfruit ripeness classification, RGB, HSV, and CIE L*a*b* color spaces were used to extract color-related features at different stages of ripeness (green, ripe, and overripe). The authors found that the CIE L*a*b* space provided better discrimination between classes due to its ability to separate lightness and color information, achieving accuracies of up to 96.2% with linear discriminant analysis (LDA) [27].

Another study on Batuan fruit grading used HSV space to assess ripeness based on the distribution of hues and saturations. This space is particularly useful for identifying changes in hues associated with ripeness or surface defects under different lighting conditions. This approach allowed fruit to be graded accurately, highlighting the utility of HSV space for handling lighting variations in uncontrolled environments [42].

In the analysis of Moroccan Date fruit varieties, 12 color channels were used, including L, a, and b, as well as others such as R, G, B, and saturation. The CIE L*a*b* space stood out for its ability to capture differences in color intensity and hues between varieties. Models based on this color space achieved an accuracy of 98% when combining textures extracted from images processed in different channels [54].

C. Classification:

The classification stage is the final component in machine vision systems. Its main objective is to assign a specific label or category to each analyzed fruit, whether based on its quality, ripeness, type, variety, or presence of defects. This stage builds on the previously extracted and processed features, using algorithms that interpret these features to make accurate and consistent predictions.

In the reviewed studies, classification is carried out using a variety of approaches, including traditional machine learning algorithms and advanced deep learning models (Table 3). These algorithms are evaluated based on metrics such as precision and accuracy, as well as their ability to handle large volumes of data in real-world scenarios. Furthermore, the selection of the classification method depends on factors such as the type of data, system characteristics, and the application context, whether in orchards, industrial processing lines, or end-use points.

1. *Classification algorithms used*

In the studies analyzed, the use of machine learning techniques stands out, with a particular emphasis on deep learning models using convolutional neural networks (CNNs). These algorithms have proven to be highly effective in addressing the classification stage in computer vision systems, thanks to their ability to learn complex and generalizable representations directly from the input data.

Pre-trained models, such as ResNet, VGG, Inception, and DenseNet, are widely used for the classification of multiple types of fruits. These models, originally designed for general image recognition tasks, are adapted through transfer learning to handle specific categories within fruit classification. Their application is particularly useful in scenarios where category-specific analysis is required, such as differentiation between fruit varieties or analysis of ripeness and defects.

Table 2 shows consolidated information on the classification methods used in each study. Other algorithms used to compare their efficiency are also described.

2. *Model accuracy and performance*

A direct comparison between all models is not possible in the reviewed studies, as each investigation uses specific datasets, capture conditions, and objectives that affect these parameters. Factors such as fruit type, image quality, lighting conditions, and pre-processing methods vary significantly, making it difficult to establish homogeneous comparisons.

Although a direct comparison between all studies is not possible due to their contextual differences, several stand out for evaluating multiple methods under the same conditions, allowing for a more accurate comparison. Some examples are presented below.

In the paper [27], the authors evaluated different classification models to determine the ripeness level of starfruit using images captured with a smartphone. These models included Linear Discriminant Analysis (LDA), Linear Support Vector Machines (SVMs), Quadratic SVM, Fine K-Nearest Neighbor (KNN), and Subspace Discriminant Analysis (SDA). The models were evaluated in terms of accuracy during calibration and validation stages (see Table 5).

In [46], the performance of several pre-trained deep learning models, adapted to a specific dataset for fruit classification, is analyzed by applying transfer learning. The evaluated models include VGG19, ResNet50, DenseNet121, DenseNet201, MobileNetV3, InceptionV3, NASNetMobile, and the proposed FruitVision model.

The FruitVision model, optimized using transfer learning on the MobileNetV3 architecture, achieved the highest accuracy on the evaluated datasets, with values between 97.96% and 99.50%, depending on the type of fruit. For example, it achieved 99.50% accuracy on bananas, outperforming DenseNet201 98.84%, which was the second-best model. In addition, FruitVision demonstrated a remarkable generalization capacity and consistency in metrics such as recall and F1-score, minimizing prediction errors. Other models, such as MobileNetV3 and ResNet101, showed good but inferior results (details in Table 6). These results highlight pre-trained models adapted through transfer learning as an efficient and accurate solution for fruit classification.

3. *Classification Objective*

In the reviewed studies, a variety of classification objectives are observed (Table 2), reflecting the specific needs of each application in artificial vision for the fruit industry. The most common objective was classification by fruit type, present in 32% of the studies, highlighting its importance for tasks such as automation in production lines and fruit recognition at points of consumption.

The second-most frequent objective was the assessment of the state of ripeness, with 23% of the studies focusing on determining levels of ripeness for applications such as automated harvesting, post-harvest quality control, and fruit selection according to commercial standards.

Finally, classification by type of variety represented 16% of the studies, a particularly relevant task for products with multiple commercially significant varieties, such as apples, dates, or mangoes, where varietal characteristics directly affect commercial value and consumer preferences.

**Figure 10:**
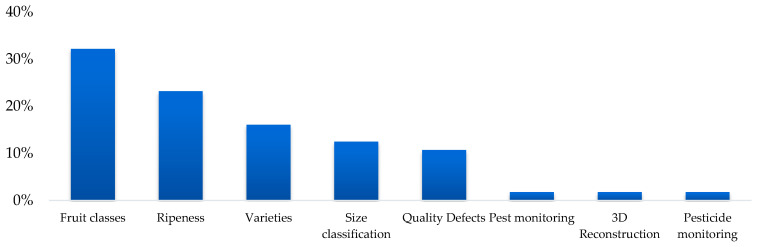
Percentage distribution of each type of application.

These objectives reflect how classification applications vary by context, from early stages of production to direct interaction with the final consumer. Figure 10 shows the percentage distribution of each type of application.

The results obtained in this systematic review highlight the diversity of approaches and objectives in the application of computer vision for the classification and evaluation of fruits. Across the categories analyzed, it was evident how acquisition, preprocessing, segmentation, feature extraction, and classification techniques are implemented and adapted according to the specific needs of each study.

4. *Techniques and algorithms*

In relation to research question RQ3, three key stages of image processing were identified that require advanced algorithms: segmentation, feature extraction, and classification. In segmentation, the standard includes techniques such as edge detection (Sobel, Canny) and thresholding methods (Otsu, mean shift clustering, watershed segmentation), which help to accurately define regions of interest by ignoring non-relevant elements of the image. It would be valuable to explore future approaches where algorithms recognize, rather than ignore, all elements present in the scene, especially in applications related to harvesting and classification at points of consumption.

In feature extraction and classification, a clear trend towards the increasing use of machine learning techniques, particularly deep learning, is evident. Pre-trained models, such as VGG-16, ResNet, and DenseNet, are commonly adapted through transfer learning to fit the specific conditions of each study, offering enhanced accuracy and adaptability across various datasets. However, the analysis of the studies highlights a lack of consensus on the optimal configurations and techniques, suggesting the need to establish common standards in the industry to improve reproducibility and performance consistency.

**Figure 11:**
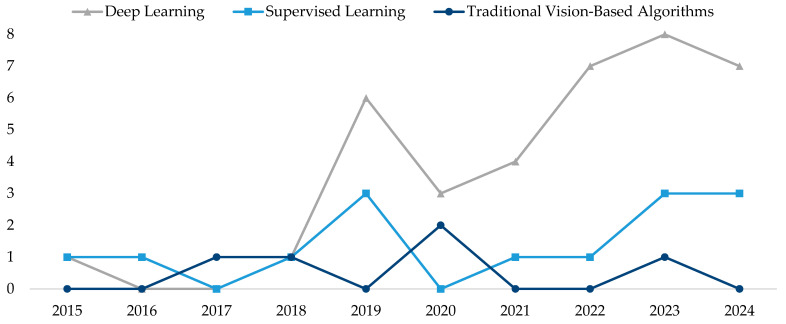
Temporal evolution of the use of different classification algorithms.

Figure 11 details the evolution of algorithm usage over time, revealing a significant shift in the field. The studies conducted between 2015 and 2024 show a steady increase in the use of deep learning methods, particularly after 2020, with a parallel decline in the reliance on traditional vision-based techniques, such as edge detection and basic histogram-based methods. For clarity, all algorithms were classified into three categories: deep learning, supervised algorithms (such as decision trees and Support Vector Machines), and traditional vision-based techniques. The results emphasize that, while deep learning currently dominates the landscape, supervised learning algorithms remain relevant in certain applications where computational resources are limited or where datasets are smaller.

## 5. Discussion

This study was designed to address key challenges in fruit sorting and inspection using machine vision. These include identifying application fields, selecting optimal hardware configurations for image capture, and analyzing the most used or best performing techniques and algorithms for fruit sorting and quality inspection.

As artificial vision systems continue to evolve, future directions in their application for agriculture, particularly in fruit inspection and classification, hold substantial promise, alongside notable challenges. One significant advancement lies in the integration of machine learning algorithms that enhance the accuracy of image analysis, allowing for real-time assessment of fruit quality and ripeness. However, the variability of fruits, influenced by diverse lighting conditions and occlusions, presents an ongoing challenge that necessitates robust adaptive algorithms. All artificial vision systems data are generated by sensors or cameras. These data are images that represent visual information in different ways. Some of the models found in the reviews were RGB, RGB-D, HSV, hyperspectral, and multispectral. From this dataset, the required characteristics must be extracted and processed according to the corresponding task. For instance, the use of mean image, color, and histogram gradients feature extraction techniques has shown promising results in classifying fruit quality [78].

Quality inspection in Industry 4.0 is crucial to integrate AI as quality control, automating the process in a more accurate and cost-effective way. Traditionally, manual inspection has been the primary method for assessing fruit quality, but this process is time-consuming, subjective, and prone to human error. Studies had explored the use of artificial vision systems for automated fruit quality inspection. These systems leverage quality attributes such as color, texture, size, shape, and the presence of defects [79].

One of the key challenges in this domain is the development of robust and accurate computer vision algorithms that can reliably detect and classify different varieties of defects for one fruit. Some studies have explored the use of multispectral imaging, including near-infrared and ultraviolet wavelengths, to enhance the detection of non-visible defects [80]. Additionally, the use of machine learning algorithms, such as convolutional neural networks, has shown promising results in the classification and grading of fruits based on their visual characteristics. These algorithms can be trained on large datasets or labeled fruit images, enabling them to learn the distinctive features associated with different quality levels and defect types.

Studies reviewed AI methods, particularly the use of CNN, commonly used for fruit sorting [17,44,46]. Several studies have explored the use of CNN for quality control, and it has been observed that in recent years, the use of CNN for fruit classification and quality inspection has significantly increased, leading to excellent results in terms of accuracy. A study proposed by [81] provides a comprehensive review of the current state of the art in CNN-based fruit classification, highlighting the key challenges, such as that the data size must be sufficiently large and well labeled to train CNN. In turn the search for model parameters and hyperparameters that are suitable to solve the specific problem, where it remains a relevant problem, as it is solved by trial-and-error adjustment until the best fits are obtained, which can be time-consuming, more so for very deep models. In turn, most of the selected quality control studies were carried out in laboratory conditions, and others were based on datasets such as Fruit 360 [19], Supermarket [15,16], and FruitNet [46]. Also, it was identified that the most used CNN architectures are the ResNet [46], VGG16 [21], VGG19 [30], and AlexNet [41]. Furthermore, MobileNet is used in studies such as [44,46], where MobileNet is more efficient with comparable effectiveness. Therefore, MobileNet may be an alternative for quality inspection in fruit processing because it involves detecting defects, ripeness levels, size classification, and sorting. Additionally, MobileNet is ideal for tasks such as (1) fast and lightweight: can run on edge devices like raspberry Pi or smartphones; (2) high accuracy: despite being lightweight, it achieves good performance in image classification; (3) real-time processing: which enables on-the-fly quality assessment; (4) pre-trained model available: can be fine-tuned using transfer learning on a fruit dataset.

**Figure 12:**
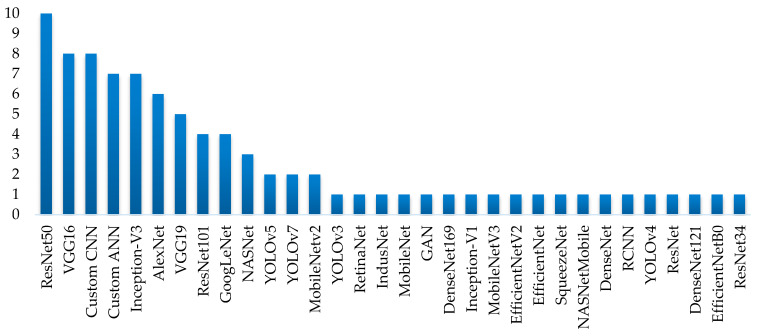
Deep learning algorithms used in the studies.

**Figure 13:**
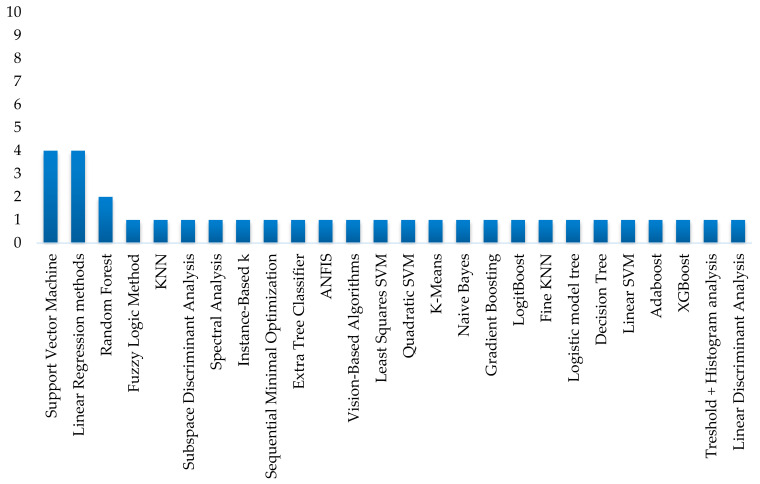
Traditional algorithms used in the studies.

For deep learning algorithms, two main categories are identified: custom models and pre-trained models adapted through transfer learning. Figure 12 provides a detailed overview of all classification models used in the reviewed studies, considering that several studies evaluate more than one model to achieve the same objective, highlighting the diversity of approaches and the continuous pursuit of optimization in each application. Similarly, Figure 13 provides, for comparison purposes, a detail of the traditional algorithms used in the analyzed studies. It is possible to see that, in this case, the predominant classification models are Support Vector Machines (SVMs) and linear regression methods.

**Figure 14:**
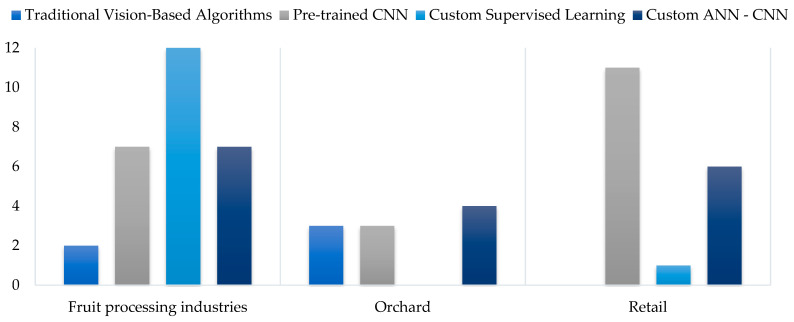
Environmental conditions of deep learning algorithms used in the studies.

Figure 12 shows that pre-trained models are predominant overall. When analyzing their usage across the three main application areas, it becomes clear that pre-trained models dominate in the retail sector and points of consumption (Figure 14), where environmental conditions, fruit types, and image capture stability are not controlled. The large amount of data incorporated into these models contributes to developing more robust and adaptable systems.

## 6. Conclusions

The present systematic literature review, guided by the PRISMA methodology, allowed us to analyze the advances in fruit sorting and quality inspection using computer vision. This study identified the main fields of application, hardware configurations, and methodologies adopted for fruit sorting using image-processing techniques. This review provides valuable guidance for future research, offering information on potential applications, the type of sensors required for image capture, the specific objectives of each application, and the techniques used in the five stages of image processing. Furthermore, the study proposes approaches that could be explored in future research, with the aim of optimizing the balance between accuracy, speed, and efficiency in industrial and commercial applications related to fruit sorting and quality inspection.

The results show new areas of application that had not been previously analyzed, such as the retail sector or final consumption points. This study also addresses in detail each of the algorithms used in the different stages of image processing, obtaining fundamental information on the techniques used for pre-processing, image segmentation, feature extraction, and classification. As demonstrated, data augmentation and transfer learning play a fundamental role in expanding the capacity of models to generalize under various conditions, providing robust performance in both industrial processing lines and retail applications.

Overall, the findings not only reinforce the state of the art but also offer new approaches that could be explored in future research, aiming to optimize the balance between accuracy, speed, and efficiency in industrial and commercial applications related to fruit sorting and quality inspection. The proposed strategies contribute to advancing the development of more scalable, accessible, and cost-effective vision systems, ensuring their adaptability in different sectors of the agricultural industry.

This review provides valuable guidance for future research, offering insights into potential applications, the type of sensors required for image capture, the specific objectives of each application, and the techniques used in the five stages of image processing. Furthermore, this study proposes approaches that could be explored in future research, with the aim of optimizing the balance between accuracy, speed, and efficiency in industrial and commercial applications related to fruit sorting and quality inspection.

## Figures and Tables

**Table 1:** Inclusion and exclusion criteria.

Inclusion/Exclusion	Criteria	
Inclusion criteria	IC1:	Studies that address inspection, classification, or detection of defects in fruits using artificial vision or image processing.
	IC2:	Articles with open access accessibility
	IC3:	Articles published between 2015 and 2024
	IC4:	Articles written in English
	IC5	Works published as scientific articles in journals (“journal articles”).
	IC6	Empirical studies presenting algorithms, image processing techniques, hardware configurations or evaluation of characteristics relevant to fruit quality.
Exclusion Criteria	EC1:	Studies that do not address fruit inspection, grading or quality or that focus on other agricultural applications with no direct relation to fruit quality control. Even if they have the search terms in the title, abstract or keywords.
	EC2:	Literature reviews, conference papers, abstracts, letters to the editor, theses, technical reports, patents or other documents that are not original scientific articles.
	EC3:	Articles written in languages other than English.
	EC4:	Articles published before 2015.
	EC5:	Papers whose full text is not available for review

**Table 2:** Summary of published selected.

Article	Year	Fruit Type	Objective	Camera Type/Lighting Source	Feature Type	Classification Method	Algorithms Used and Compared	Target
[14]	2018	Oranges	Varieties	GigE industrial camera /RGB/controlled artificial	Texture, Color, Shape	ANN and metaheuristic	Custom ANN	Fruit-processing industries
[15]	2022	Multiple fruits	Fruit classes	RGB/ambient lighting	Shape, Texture, Color	RNN	Adam with DenseNet169	Retail
[16]	2023	Multiple fruits	Fruit classes	RGB/ambient lighting	Color, Shape, Texture	CNN	VGG-16 with Spiral Optimization	Retail
[17]	2024	Mango	Varieties	Smartphone/ambient lighting	Deep Features	Cubic SVM	MobileNet-v2	Retail
[18]	2023	Multiple fruits	Fruit classes	RGB/ambient lighting	Size, Shape	CNN	YOLOv5	Fruit-processing industries
[19]	2019	Apple	Fruit classes	RGB/ambient lighting	Color, Shape, Size	CNN	ResNet50	Fruit-processing industries
[20]	2019	Olive	Varieties	24Mpx CCD/HSV/controlled artificial	Size, Mass	-	Linear Regression methods	Fruit-processing industries
[21]	2024	Banana	Ripeness	RGB/ambient lighting	Color, Texture	CNN	ResNet 34, ResNet 101, VGG16, VGG19	Fruit-processing industries
[22]	2015	Apple	Size classification	RGB/controlled artificial	Mass, Size	Fuzzy neural network	ANFIS + Linear Regression methods	Fruit-processing industries
[23]	2023	Blackberry	Ripeness	Multispectral/ambient lighting	Ripeness Stage	CNN	Custom CNN, ResNet50	Orchard
[24]	2018	Oil palm fruit	Ripeness	RGB/ambient lighting	Color, Texture, Size	Fuzzy Logic Method	Fuzzy Logic Method	Orchard
[25]	2024	Chili	Ripeness	Smartphone/ambient lighting	Color, Texture, Size	CNN	EfficientNetB0, VGG16, ResNet50	Fruit-processing industries
[26]	2016	Kiwifruit	Shape	RGB/ambient lighting	Shape, Size	-	Linear Regression methods	Fruit-processing industries
[27]	2023	Starfruit	Ripeness	Smartphone/controlled artificial	Color Space Model	LDA, KNN, SVM	Linear Discriminant Analysis (LDA), Linear Support Vector Machine, Quadratic SVM, Fine KNN, Subspace Discriminant Analysis	Fruit-processing industries
[28]	2019	Date fruit	Ripeness	RGB/ambient lighting	Color, Texture, Size	CNN	AlexNet, VGG16	Orchard
[29]	2020	Date fruit	Shape	RGB/ambient lighting	Color, Shape	Vision-Based Algorithms	-	Fruit-processing industries
[30]	2020	Date fruit	Ripeness	RGB/ambient lighting	Texture, Color, Shape	CNN, SVM	ResNet, VGG-19, Inception-V3, NASNet, SVM	Orchard
[31]	2021	Multiple fruits	Fruit classes	RGB/ambient lighting	Color, Shape, Texture	CNN	VGG-19 + Pyramid histogram of oriented gradient (PHOG)	Retail
[32]	2024	Multiple fruits	Fruit classes	RGB/ambient lighting	Texture, Shape, Color	ANN, CNN	Custom ANN, AlexNet, Squeezenet, GoogLeNet, ResNet50	Orchard
[33]	2020	Carrot	Shape	RGB/controlled artificial	Length, Diameter, Shape	Vision-Based Algorithms	-	Fruit-processing industries
[34]	2023	Cerasus Humilis	Quality Defects	Hyperspectral/Controlled artificial	Defects	LS-SVM	Least Squares–Support Vector Machine	Fruit-processing industries
[35]	2024	Shaanxi Plum	Fruit classes	RGB/ambient lighting	Variety, Wax Bloom	CNN	RetinaNet, Faster R-CNN, YOLOv3, YOLOv5, YOLOv7	Fruit-processing industries
[36]	2024	Mango	Shape	HSV/controlled artificial	Shape, Surface Defects	KNN, DT, RF, ADB, XGB, GB, ET, SVM.	XGBoost, Random Forest, Extra Tree Classifier, Gradient Boosting, SVM, Adaboost, Decision Tree, KNN	Fruit-processing industries
[37]	2019	Multiple fruits	Fruit classes	RGB/ambient lighting	Shape, Texture	CNN	Alexnet, GoogLeNet	Retail
[38]	2020	Multiple fruits	Fruit classes	RGB/ambient lighting	Contrast Enhanced Features	CNN + RNN	Custom CNN	Orchard
[39]	2015	Multiple fruits	Fruit classes	RGB/ambient lighting	Wavelet-Entropy	FNN	Feed-Forward Neural Network	Retail
[40]	2021	Multiple fruits	Fruit classes	RGB/ambient lighting	Residual Features	SVM	SVM, DT, Forest, KNN.	Retail
[41]	2023	Multiple fruits	Fruit classes	RGB/controlled artificial	Adversarial Robust Features	CNN	AlexNet, GoogLeNet, ResNet101, VGG16	Retail
[42]	2019	Batuan	Quality Defects	RGB/ambient lighting	Depth, Shape	SVM	SVM	Fruit-processing industries
[43]	2022	Apple	Fruit classes	RGB/ambient lighting	Color, Size	CNN + RNN + LSTM	CNN + RNN + LSTM	Orchard
[44]	2022	Multiple fruits	Fruit classes	RGB/ambient lighting	Enhanced Features	CNN	IndusNet, VGG16, MobileNet	Retail
[45]	2022	Multiple fruits	Fruit classes	RGB/ambient lighting	Shape, Color	CNN	YOLOv7, ResNet50, VGG16	Retail
[46]	2024	Multiple fruits	Fruit classes	RGB/ambient lighting	Texture, Size, Color	CNN	FruitVision-(MobileNetV3, VGG19, ResNet50, Resnet101, DenseNet121, DenseNet201, InceptionV3, NASNetMobile)	Retail
[47]	2021	Banana	3D Reconstruction	RGB/mixed	3D Volumetric Features	GAN	GAN	Retail
[48]	2022	Multiple fruits	Ripeness	RGB/ambient lighting	Color, Texture	CNN	Alexnet, VGG, GoogLeNet, Resnet	Retail
[49]	2023	Grapes	Pest monitoring	Hyperspectral/Controlled artificial	Hyperspectral Features	Spectral Analysis	Spectral Analysis	Orchard
[50]	2021	Indian Jujube	Varieties	RGB/ambient lighting	Morphological, Color	ANN	Custom ANN	Fruit-processing industries
[51]	2020	Date fruit	Ripeness	RGB/ambient lighting	Maturity Indicators	CNN	VGG-19, NASNet, Inception-V3	Orchard
[52]	2019	Mango	Quality Defects	RGB/Controlled artificial	Color, Size	KNN	KNN	Fruit-processing industries
[53]	2022	Multiple fruits	Ripeness	RGB/ambient lighting	Freshness Attributes	CNN	YOLOv4	Retail
[54]	2023	Date fruit	Varieties	RGB/Controlled artificial	Texture Features	SMO, Naive Bayes, Ibk, LogitBoost, LMT	SMO, Naive Bayes, Ibk, LogitBoost, LMT	Fruit-processing industries
[55]	2023	Hawthorn Fruit	Ripeness	RGB/Controlled artificial	Color, Ripeness	CNN	Custom CNN, Inception-V3, ResNet50	Fruit-processing industries
[56]	2024	Pomegranate	Quality Defects	RGB/Controlled artificial	Sunburn Features	ANN, SVM	ANN, SVM	Fruit-processing industries
[57]	2024	Custard apples	Ripeness	RGB/ambient lighting	Color, Areole Opening	SVM, KNN	SVM, K-Means	Fruit-processing industries
[58]	2021	Multiple fruits	Varieties	RGB/Mixed	Enhanced Features	CNN	Inception-V3	Retail
[59]	2019	Multiple fruits	Object detection	RGB/Mixed	Shape, Color	CNN	Custom CNN	Retail
[60]	2019	Pomegranate	Size classification	RGB/ambient lighting	Weight, Size	ANN	Custom ANN	Fruit-processing industries
[61]	2024	Multiple fruits	Varieties	RGB/Mixed	Visual and Textural	CNN	EfficientNetV2	Retail
[62]	2022	Apple	Quality Defects	Hyperspectral/Controlled Artificial	Spectral and Spatial	RF	Random Forest	Fruit-processing industries
[63]	2018	Olive	Size classification	RGB/Controlled Artificial	Size, Mass	-	Linear Regression methods	Fruit-processing industries
[64]	2019	Olive	Varieties	RGB/Controlled Artificial	Variety	CNN	Inception—ResNetV2 (AlexNet, InceptionV1, InceptionV3, Resnet-50, ResNet-101)	Fruit-processing industries
[65]	2017	Apple	Pesticide monitoring	Multispectral/Controlled Artificial	Spectral Features	Vision-Based Algorithms	Threshold + Histogram analysis	Orchard
[66]	2022	Apple	Ripeness	RGB/Controlled Artificial	Appearance, Freshness	CNN	ResNet, DenseNet, MobileNetV2, NASNet, EfficientNet	Fruit-processing industries
[67]	2023	Banana	Quality Defects	RGB/ambient lighting	Color, Texture, Size	CNN	KEGCNN (Knowledge Embedded-Graph CNN	Fruit-processing industries
[68]	2015	Multiple fruits	Fruit classes	RGB/ambient lighting	Signal Parameters (S11, S21)	KNN, ANN	KNN, ANN	Fruit-processing industries
[69]	2023	Multiple fruits	Fruit classes		Variety	CNN	Custom CNN	Retail
[70]	2023	Olive	Varieties	RGB/ambient lighting	Variety	CNN	TinyML approach	Fruit-processing industries

**Table 3:** Benchmark datasets used in the articles under study.

Reference	Dataset Name	Images Used	Dataset Reference
[15,16]	Supermarket produce	2633	[71]
[17]	Mango Variety	1853	[72]
[19]	Fruit 360	8271	[73]
[30]	Date fruit dataset for automated harvesting and visual yield estimation	8079	[74]
[31]	Fruit 360	65,429	[73]
[32]	Fruit 360	8072	[73]
[40]	Fruit 360	22,688	[73]
[45]	FIDS-30	971	[75]
[46]	FruitNet	19,526	[76]
[51]	Date fruit dataset for automated harvesting and visual yield estimation	8079	[74]
[61]	FruitNet	14,700	[76]
[66]	Internal feeding-worm database of the comprehensive automation for specialty crops	8791	[77]

**Table 4:** Percentage distribution by image capture device.

Capture Device	Quantity	Reference
RGB Camera	84%	[14,15,16,18,19,20,21,22,24,26,28,29,30,31,32,33,35,36,37,38,39,40,41,42,43,44,45,46,47,48,50,51,52,53,54,55,56,57,58,59,60,61,63,64,66,67,70]
Hyperspectral	5%	[34,49,62]
Smartphone	5%	[17,27]
Multispectral	4%	[23,65]
Radio frequency	2%	[69]

**Table 5:** Comparative results between different types of classification methods [27].

Classification Model	Accuracy of Classification (%)	Precision of Validation (%)
Linear Discriminant Analysis (LDA)	96.2	93.3
Linear SVM	88.7	86.7
Quadratic SVM	90.3	86.7
Fine KNN	94.3	93.3
Subspace Discriminant Analysis (SDA)	90.4	86.7

**Table 6:** Comparative results between different types of classification methods and the method FruitVision proposed by [27] for bananas.

Model	Accuracy (%)	Precision (%)	Recall (%)	F1 Score (%)	Specificity (%)
VGG19	97.54 ± 0.17	97.25 ± 0.34	96.85 ± 0.21	97.05 ± 0.48	96.12 ± 0.21
ResNet50	96.21 ± 0.62	96.42 ± 0.27	96.05 ± 0.68	96.23 ± 0.30	95.74 ± 0.38
ResnNet101	98.30 ± 0.04	97.66 ± 0.31	96.94 ± 0.37	97.30 ± 0.49	95.76 ± 0.62
DenseNet121	98.42 ± 0.33	97.21 ± 0.28	97.14 ± 0.24	97.17 ± 0.33	96.27 ± 0.84
DenseNet201	98.84 ± 0.28	98.35 ± 0.45	97.51 ± 0.29	97.93 ± 0.67	97.10 ± 0.36
MobileNetV3	97.24 ± 0.43	96.82 ± 0.36	96.83 ± 0.47	96.82 ± 0.31	96.88 ± 0.22
InceptionV3	94.11 ± 0.57	94.21 ± 0.65	93.64 ± 0.62	93.92 ± 0.57	93.27 ± 0.26
NASNetMobile	96.74 ± 0.27	96.72 ± 0.38	96.25 ± 0.34	96.48 ± 0.63	96.38 ± 0.65
**FruitVision (Proposed)**	**99.50 ± 0.20**	**99.19 ± 0.28**	**98.88 ± 0.74**	**99.03 ± 0.55**	**98.77 ± 0.34**
